# User Behaviors and User-Generated Content in Chinese Online Health Communities: Comparative Study

**DOI:** 10.2196/19183

**Published:** 2021-12-15

**Authors:** Yuqi Lei, Songhua Xu, Linyun Zhou

**Affiliations:** 1 Institute of Medical Artificial Intelligence The Second Affiliated Hospital of Xi’an Jiaotong University Xi’an China

**Keywords:** online health community, user behaviors, user-generated content, social network analysis, weighted knowledge network

## Abstract

**Background:**

Online health communities (OHCs) have increasingly gained traction with patients, caregivers, and supporters globally. Chinese OHCs are no exception. However, user-generated content (UGC) and the associated user behaviors in Chinese OHCs are largely underexplored and rarely analyzed systematically, forfeiting valuable opportunities for optimizing treatment design and care delivery with insights gained from OHCs.

**Objective:**

This study aimed to reveal both the shared and distinct characteristics of 2 popular OHCs in China by systematically and comprehensively analyzing their UGC and the associated user behaviors.

**Methods:**

We concentrated on studying the lung cancer forum (LCF) and breast cancer forum (BCF) on Mijian, and the diabetes consultation forum (DCF) on Sweet Home, because of the importance of the 3 diseases among Chinese patients and their prevalence on Chinese OHCs in general. Our analysis explored the key user activities, small-world effect, and scale-free characteristics of each social network. We examined the UGC of these forums comprehensively and adopted the weighted knowledge network technique to discover salient topics and latent relations among these topics on each forum. Finally, we discussed the public health implications of our analysis findings.

**Results:**

Our analysis showed that the number of reads per thread on each forum followed gamma distribution (*H_L_*=0, *H_B_*=0, and *H_D_*=0); the number of replies on each forum followed exponential distribution (adjusted *R_L_*^2^=0.946, adjusted *R_B_*^2^=0.958, and adjusted *R_D_*^2^=0.971); and the number of threads a user is involved with (adjusted *R_L_*^2^=0.978, adjusted *R_B_*^2^=0.964, and adjusted *R_D_*^2^=0.970), the number of followers of a user (adjusted *R_L_*^2^=0.989, adjusted *R_B_*^2^=0.962, and adjusted *R_D_*^2^=0.990), and a user’s degrees (adjusted *R_L_*^2^=0.997, adjusted *R_B_*^2^=0.994, and adjusted *R_D_*^2^=0.968) all followed power-law distribution. The study further revealed that users are generally more active during weekdays, as commonly witnessed in all 3 forums. In particular, the LCF and DCF exhibited high temporal similarity (*ρ*=0.927; *P*<.001) in terms of the relative thread posting frequencies during each hour of the day. Besides, the study showed that all 3 forums exhibited the small-world effect (mean *σ_L_*=517.15, mean *σ_B_*=275.23, and mean *σ_D_*=525.18) and scale-free characteristics, while the global clustering coefficients were lower than those of counterpart international OHCs. The study also discovered several hot topics commonly shared among the 3 disease forums, such as disease treatment, disease examination, and diagnosis. In particular, the study found that after the outbreak of COVID-19, users on the LCF and BCF were much more likely to bring up COVID-19–related issues while discussing their medical issues.

**Conclusions:**

UGC and related online user behaviors in Chinese OHCs can be leveraged as important sources of information to gain insights regarding individual and population health conditions. Effective and timely mining and utilization of such content can continuously provide valuable firsthand clues for enhancing the situational awareness of health providers and policymakers.

## Introduction

### Background

An online community is a social group created by internet users for a variety of purposes and interests. The rapid development of the “internet plus” [1] technology has further promoted the value of online communities in recent years. In the meanwhile, the health consciousness of populations and their motivation for better self-health management have been steadily growing. Propelled by the strong desire to mitigate the information asymmetry between doctors and patients pervasive in traditional health care communications, patients have gained a new way to share their disease situation and receive needed health care advice through online health communities (OHCs). For example, a continuously rising number of patients and their relatives continually participate in OHCs, actively share their treatment experiences, and openly express their personal opinions and feelings on various issues encountered during treatment or the whole care journey. The value of OHCs for exchanging emotional communications and delivering social support for patients and their families has also been widely recognized [2]. At present, an increasing multitude of user-generated content (UGC) and associated online user behaviors are becoming available on OHCs. The US Office of the National Coordinator for Health Information Technology defines patient-generated health data as health-related data created, recorded, or gathered by or from patients (or family members or other caregivers) to help address a health concern [3]. The Chinese National Health Commission publicly released an Action Plan for the Further Improvement of Medical Services (2018-2020), which emphasizes the role of patient organizations in knowledge sharing, whole-course disease management, rehabilitation support, drug development, and clinical trials [4]. However, such UGC in Chinese OHCs and their associated user behaviors are often underexplored and rarely analyzed systematically, thus losing valuable clues and evidence for improving treatment design and patient care.

### Status of Research Concerning OHCs

A number of OHCs exist, providing users with diverse and fluent ways to exchange information, share experiences, seek answers, and receive support. PatientsLikeMe is the first and also the largest social network platform in the world dedicated to patients. By 2018, more than 650,000 users had communicated and shared their health information over the platform, with more than 2900 diseases involved [5]. MyHealthTeams [6] is a social network for people living with chronic diseases, which aims to provide mutual aids for its participants, and has gathered more than 2 million users spread over 33 online disease platforms. Several OHCs, such as Breastcancer [7] and BecomeAnEX [8], are also popular among patients. In China, Manyoubang [9] provides an interactive OHC for patients with chronic diseases, which has more than 22 subforums. Yi Xiang Network [10] is the largest case sharing website in China, providing services, such as case upload, communications, and mutual assistance, for patients. Mijian [11] is the largest interactive OHC for patients in China at the time of writing this manuscript, which integrates multiple single disease forums with interactive question and answer functions. Other OHCs in China, such as Sweet Home [12] and Lymphoma Home [13], mainly focus on servicing patients with chronic conditions. Meanwhile, in some general-purpose Chinese online forums (eg, Tianya [14], Tieba [15], and Zhihu [16]), there are also subforums specifically dedicated to disease-centric discussions.

Since the emergence of versatile OHCs, scholars have attempted to analyze these virtual communities from various perspectives. For example, Smailhodzic et al [17] conducted a literature review covering 22 articles, according to which, patients’ use of social media were classified into the following 6 categories: emotional, information, esteem, network support, social comparison, and emotional expression. Dongxiang [18] overviewed OHCs in China from 3 perspectives, including their UGC, and the characteristics of participants and underlying online communities. Wu et al [19] summarized research hotspots concerning OHCs and the evolution of OHCs, as well as the key analysis methods for OHCs. To reveal factors that may motivate knowledge sharing in OHCs, scholars utilized text mining to better understand and predict user participation [20,21]. Fernandes et al [22] adopted a netnography method to analyze the positive impact of OHCs on the prognosis of diabetes. Li [23] utilized the structural equation model to study factors affecting individual patient’s willingness to share medical information. Scholars also studied the distribution of health topics according to questions and answers on OHCs using machine learning approaches [24-27]. In addition, scholars utilized social network analysis methods to analyze knowledge exchange behaviors among users in OHCs by constructing and examining underlying knowledge-sharing networks [28-33].

Overall, existing research on OHCs has mainly focused on uncovering users’ motivation for participating in the OHCs, discussing factors affecting users’ online knowledge-sharing behaviors, and mining UGC in OHCs. For Chinese OHCs, existing research primarily concentrates on examining small-scale single-disease forums. In comparison with peer international studies, both the breadth and depth of current analysis regarding Chinese OHCs are much more limited, calling for expanded efforts to broaden the understanding and strengthen preliminary findings produced through existing studies. To meet the demand and fill the gap, this study comprehensively examined 3 representative disease forums hosted on the 2 most popular Chinese OHCs. The large-scale evaluation reveals both the shared traits and distinct characteristics of user behaviors and UGC on these forums to shed light on understanding user behaviors and UGC on Chinese OHCs in general.

### Objectives

Given the popularity and proliferation of OHCs, understanding multifaceted patient experiences reflected from UGC in these forums and related user behaviors can provide many valuable insights for enhancing public health awareness and improving the quality of the care delivered. Comprehensive and in-depth analysis of such user content and behaviors can also help optimize the design and management of OHCs from a software engineering perspective, as well as the design and development of better community-based knowledge services at large. Driven by the above anticipated benefits, this study performed an in-depth analysis on UGC and related online user behaviors in 3 large-scale OHCs in China. We utilized a variety of social network analysis methods and constructed a knowledge-sharing network for each OHC to study the evolution of OHCs, discover characteristics of user behaviors, uncover salient topics and their relations in each of the virtual communities, and reveal common traits and distinct characteristics in the 3 OHCs examined. Through these case analyses, we also aimed to offer insights regarding user behaviors and UGC in Chinese OHCs in general.

## Methods

### Data Collection

Two influential OHCs in China, Mijian and Sweet Home, were selected for analysis in this study. Mijian was selected for analysis in this study because it is the largest OHC for patients in China at present. The site targets to serve patients diagnosed with chronic, severe, or rare diseases, aiming to help relieve their psychological stresses, learn disease-related health knowledge, and effectively acquire medical resources. Sweet Home was selected for analysis in this study because it is the largest OHC in China for patients with diabetes. The site offers categorized forums for medical consultation, service guidance, and emotional expression. Through the site, patients with diabetes can not only discuss their medical conditions, but also connect and communicate remotely with other patients across the country. Regarding the 2 focus OHCs identified in this study, our analysis concentrated on examining the lung cancer forum (LCF) and breast cancer forum (BCF) on Mijian, and the diabetes consultation forum (DCF) on Sweet Home in particular because of their predominant popularity among users of the 2 OHCs and the significance of the 3 diseases for the well-being of Chinese patients and the population as a whole given that breast cancer and lung cancer represent the 2 leading chronic noncommunicable diseases in the world and China has the largest number of patients with diabetes globally [34,35].

In a disease forum, a single conversation is referred to as a “thread” (ie, a topic). Users can respond to another person’s thread, which is referred to as a “reply.” Thus, a post made by a user on a forum can either be an original thread created by the user or a reply to another user’s thread [36]. We crawled all threads on the 3 focus disease forums from their respective forum establishment dates (LCF: November 15, 2013; BCF: August 25, 2015; DCF: September 1, 2005) to October 20, 2020. Once a post was crawled, we also obtained its ID, posting time, number of reads, and number of replies. We then performed a series of data cleaning operations, including filtering posts automatically created by chatbots on these forums and deleting missing data. Table 1 presents key statistics of the acquired online data sets in comparison with those of data sets used in peer studies [20,24,26,28,30,37,38], which shows that the scale of the current analysis significantly transcends that of all prior efforts.

**Table 1:** Comparison between experimental data sets analyzed in this study and the counterpart data sets in peer studies.

Study	Website	Forum	Number of threads	Number of users	Number of replies
Present study	Mijian [11]	Lung cancer forum	37,090	22,610	254,687
Present study	Mijian [11]	Breast cancer forum	112,790	31,909	2,123,728
Present study	Sweet Home [12]	Diabetes consultation forum	41,060	26,751	466,225
Wu et al [28]^a^	Yi Xiang Network [13]	Breast cancer forum	754	540	3498
Wu et al [37]^a^	39 Health Network [39]	Hepatitis B forum	1066	N/A^c^	N/A
Wu et al [38]^a^	Tieba [15]	Tumor forum	2009	1476	11,940
Shi et al [30]^a^	Manyoubang [9]	Diabetes mutual aid forum	777	636	3553
Wang et al [20]^b^	Breastcancer [7]	Breast cancer forum	107,549	49,552	2,800,000
Wang et al [24]^b^	BecomeAnEX [8]	Smoking cessation	38,156	5435	316,886
Della Rosa et al [26]^b^	Facebook [40]	Multiple sclerosis	N/A	24,915	N/A

^a^Online health communities in China.

^b^Online health communities in other countries.

^c^N/A: not applicable.

### Social Network Analysis

A social network is a social structure made up of a set of social actors (such as individuals and organizations), sets of dyadic ties, and other social interactions between actors [41]. Social network analysis can help identify community structures at the network level, as well as individual behaviors at the single-user level. Since a user could be both a thread author and a reply author, this study adopted a directed network structure to model the community network. In such a directed network, each edge of the network is directional, where the in-degree of a node refers to the number of directed edges ending with the node. Conversely, the out-degree of a node is the number of directed edges starting from the node. The total degree of a node is the total number of its network neighbors irrespective of the tie direction (ie, the sum of its in-degree and out-degree).

We conducted a topological analysis for complex networks [41] to explore the structural characteristics of each forum. Our analysis was carried out in 2 steps. First, we explored the small-world effect of each social network. We took the path length between 2 nodes as the minimum number of edges connecting these nodes in the network. The average path length, also known as characteristic path length, is defined as the average number of steps along the shortest paths for all possible pairs of network nodes. Let *d_ij_* denote the shortest distance between 2 nodes *i* and *j* in the network. Assume that *d_ij_*=0 if *i*=*j* or *j* cannot be reached from *i*. Then, the average path length *L* is as follows:



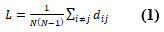



where *N* is the number of network nodes. The clustering coefficient of a network measures the degree of node clustering in the network. Assume a node *k* has *n* adjacent neighboring nodes (*N*_1_, *N*_2_, …, *N*_n_). If 2 nodes *i* and *j* are connected with a link, the directed link is denoted as *e_ij_*. The local clustering coefficient of the node *k* is defined as follows:



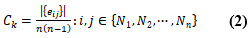



Assume the entire network has *K* nodes in total. The average clustering coefficient of the network is the mean of the clustering coefficients of all its nodes, that is,

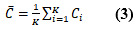



The small-world effect, also known as the 6 degrees of separation, is the idea that all strangers can be related through 6 or fewer people [42]. Watts et al proposed a small-world network model (WS model) [43], in which a small-world network is characterized by a small average path length and a high clustering coefficient. As a general method for quantifying the small-world effect of a network, the network can be measured by comparing its clustering coefficient and average path length with those of an equivalent Erdös–Rényi (ER) random network that has the same number of nodes and edges [44]. To construct such an equivalent random network, we employed the following generative procedure: Let N and M be the number of nodes and edges expected of the network to be generated, respectively. The network is initialized to have N unconnected nodes. At each step, we randomly selected and linked a pair of nodes not currently connected in the network. We repeated the above step until all M edges were added into the network. Given the random network generated, *σ* can be calculated as follows:



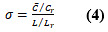



where 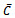 is the average clustering coefficient of the network, *L* is the average path length of the network, *C_r_* is the average clustering coefficient of the equivalent ER random network, and *L_r_* is the average path length of the equivalent ER random network. If *σ*>1 (eg, 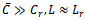), the network is considered a small-world network [44], in which case, it is assumed that knowledge can be spread efficiently and rapidly in the community represented by the network.

Second, we explored the scale-free property of social networks. Scale-free property is a structural characteristic concerning a network as introduced by Barabási et al [45]. Across scientific domains and classes of networks, it is common to encounter the claim that most or all real-world networks are scale free. Generally, a network is deemed scale free if the fraction of nodes with degree *k* follows a power-law distribution *P*(*k*)=*c*×*k*^−^*^r^*, where *r*>1. The property mainly comprises the following 2 aspects. First, the distribution of nodes follows a power-law distribution, where most nodes have few links, while a small fraction of nodes has a large number of links. In a power-law distribution, the probability that a node has degree *k* follows the distribution equation *P*(*k*)=*c*×*k*^−^*^r^*, where *c* and *r* are network-specific constants. It is generally believed that if the degree of nodes in a network follows a power-law distribution, the network is a scale-free network. Second, during a network growth process, new nodes preferentially establish relations with well-connected nodes. The scale-free network model is also commonly referred to as the B-A model [45].

### Weighted Knowledge Network

Topic analysis techniques can be leveraged to extract conceptual topics, determine their types, and analyze their internal structures latent in a large text corpus. In this study, we analyzed health topics on OHCs to identify hot topics and the salient health information needs of their users.

We executed the topic analysis in 2 steps. First, we extracted key phrases among UGC according to point-wise mutual information (PMI), as well as the left and right information entropy, with which the co-occurrence relationship between words can be efficiently found. In this step, mutual information is mainly used to measure the degree of correlation between 2 signals according to information theory [46], which is repurposed to measure the degree of interdependence between 2 variables. In natural language processing (NLP), PMI is used to calculate the degree of correlation between 2 words, so that the co-occurrence of words can be found from a statistical perspective to examine whether any semantic correlation or thematic correlation exists between a pair of words. The PMI of 2 adjacent words *x* and *y* is computed as follows:



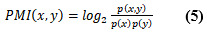



where *p*(*x*) is the probability of word *x* appearing in all threads, that is, *p*(*x*)=the number of occurrences of word *x* / the total number of words in all threads; *p*(*y*) is the probability of word *y* appearing in all threads, that is, *p*(*y*)=the number of occurrences of word *y* / the total number of words in all threads; and *p*(*x*,*y*) is the joint probability of *x* and *y*, which is the probability that 2 words (*x*,*y*) appear adjacent to each other in the text. A higher PMI of *x* and *y* is associated with higher internal aggregation and greater possibility of the 2 words forming a phrase. Conversely, those 2 words are more likely to have phrasal boundaries.

Entropy is an uncertainty measure associated with a random variable. A higher entropy is associated with greater underlying information content and hence higher uncertainty [47]. In NLP, the left and right entropy of the word *W* are defined as follows:



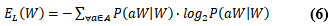





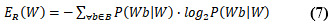



where *E_L_* and *E_R_* are the left entropy and right entropy of the word *W*, respectively; *A* and *B* represent the sets of all words appearing to the left and right of *W*, respectively; and *a* and *b* represent the words appearing immediately on the left and right sides of *W*, respectively. Greater left or right entropy is associated with a higher degree of freedom of the word, which indicates more abundant choices for a target word surrounding the given word *W*.

Second, we treated a keyword as a node and the co-occurrence relationship between a pair of keywords as an edge to construct a weighted knowledge network (WKN) [37,48]. In the process, we also assigned weights to the nodes and edges according to weights of the corresponding keywords and the relationship strength between the corresponding key phrases. The WKN integrated and modeled fragmentation knowledge of the thematic content, which can be used to effectively discover the internal relationships and overall characteristics of a knowledge network. More specifically, we summed the PMI value of the left entropy and right entropy calculated above, which was used as a measure of 2 words as a phrase. We then extracted key phrases in each post and their respective weights where the frequency of a key phrase is used as the weight of the key phrase. We define *E* as the keywords co-occurrence set and *Q*(*E*) as the weight set associated with *E*, as follows:



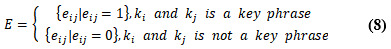





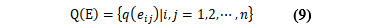



In the above equations, if keywords *k_i_* and *k_j_* formulate a key phrase, as indicated by a co-occurrence relationship between them in the WKN, then *e_ij_=*1; otherwise, *e_ij_=*0. *q*(*e_ij_*) is the weight of *e_ij_*. *q*(*e_ij_*)=*n*(*e_ij_*)/*N*, where *n*(*e_ij_*) is the number of occurrences of a key phrase in all phrases and *N* is the total number of all phrases. Next, all detected key phrases were organized as a keywords set *K*, for which an associated keyword weight set *Q*(*K*) was introduced as follows:



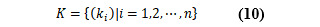





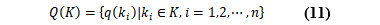



where *k_i_* is a keyword and *q*(*k_i_*) is its weight. *q*(*k_i_*)=*m*(*k_i_*)/*M*, where *m*(*k_i_*) is the number of occurrences of a keyword *k_i_* in the 200 key phrases and *M* is the total number of all keywords in the 200 key phrases.

Now, we can define a WKN model for the concerned OHC as follows:



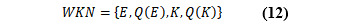



According to the constructed WKN model, the results can be displayed by social network visualization tools.

## Results

### Descriptive Statistics

Table 2 presents descriptive statistics of the OHC data analyzed in this study where extreme outliers were removed during the preprocessing. In terms of the number of reads and replies per thread, the data distribution was severely nonuniform. In other words, most of the threads had fewer reads and replies, and only a few threads got a large number of reads and replies, which meant that most users preferentially read the threads that had received a larger number of replies, thus resulting in the polarization. The SD is a measure of the amount of variation or dispersion of a set of numbers, which is also affected by the volume of data analyzed. The coefficient of variation is a statistical measure of the dispersion of data points around the mean of a data series. The coefficient of variation of the general normal distribution is less than 1. Considering the large coefficient of variation for each attribute listed in Table 2 and according to the skewness of the frequency distribution graph (*SK_L_*=35.41, *SK_B_*=25.65, and *SK_D_*=12.45), we concluded that none of them follows a normal distribution.

**Table 2:** Statistical characteristics of the 3 data sets analyzed in this study.

Data set and variable		Minimum	Q1	Median	Q3	Maximum	Mean	SD	CV^a^
**Lung cancer forum**									
	Reads	15	362	758	2043.5	98,050	531.25	1136.88	2.14
	Replies	0	4	8	16	3405	15.34	31.98	2.08
	Followers	0	5	14	49	10,127	54.52	456.76	8.38
	Threads	1	1	2	5	595	5.84	20.12	3.44
**Breast cancer forum**									
	Reads	14	297	504	854	90,783	368.81	719.18	1.95
	Replies	0	8	15	26	1017	21.47	23.57	1.08
	Followers	0	15	46	166	2627	219.26	474.06	2.16
	Threads	1	1	3	12	5118	43.34	317.59	4.53
**Diabetes consultation forum**									
	Reads	38	812	1203	1813	95,905	1065.60	1342.66	1.26
	Replies	0	4	8	14	796	11.44	14.99	1.31
	Followers	0	0	0	0	466	1.10	7.63	6.93
	Threads	0	2	4	14	3862	20.60	94.14	4.57

^a^CV: coefficient of variation; CV=SD/mean.

**Figure 1:**
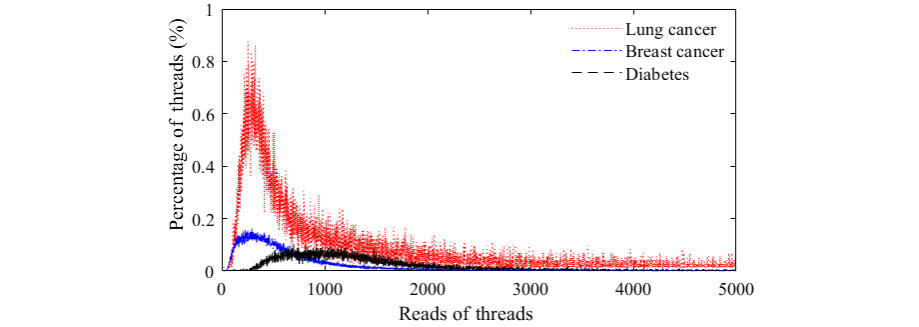
The distribution of the number of reads per thread in each of the forums (lung cancer, breast cancer, and diabetes consultation). For better visualization, the horizontal axis only shows the number of reads per thread up till 5000, since such threads hardly exist.

**Figure 2:**
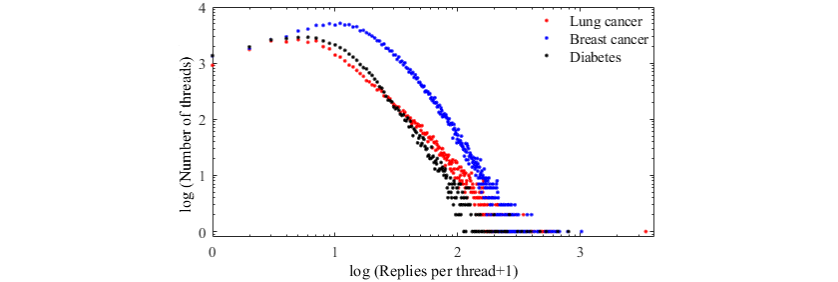
The log-log distribution of the number of replies per thread in each of the forums (lung cancer, breast cancer, and diabetes consultation).

Next, we plotted the frequency distribution of the number of reads per thread in each forum, as shown in Figure 1. We also adopted the K-S test (*H_L_*=0, *H_B_*=0, and *H_D_*=0), from whose results we can determine that the number of reads per thread follows the gamma distribution [49]. When we plotted the frequency distribution of the number of replies per thread, we found that they exhibited an obvious long-tail phenomenon, which suggests that the number of replies per thread follows the power-law distribution. We further found that the log-log distribution of the number of replies per thread was noticeably curved, as shown in Figure 2, according to which we determined that the number of replies per thread follows an exponential distribution. The 
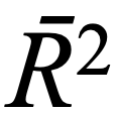
 values of all fitted exponential curves were above 0.94 (
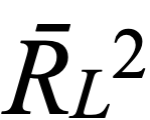
=0.946, 
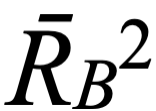
=0.958, and 
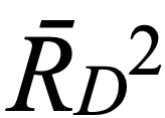
=0.971). Through similar analysis procedures, we further found that both the number of threads a user is involved with (
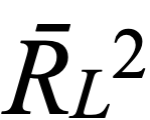
=0.978, 
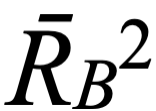
>=0.964, and 
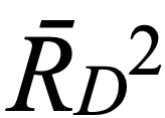
=0.970) and the number of followers of a user (
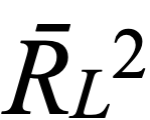
>=0.989, 
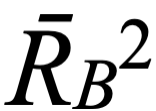
=0.962, and 
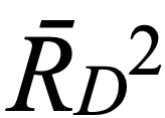
=0.990) followed the power-law distribution, and the 
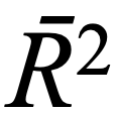
 values of all fitted power-law curves were above 0.96.

### User Activities

**Figure 3:**
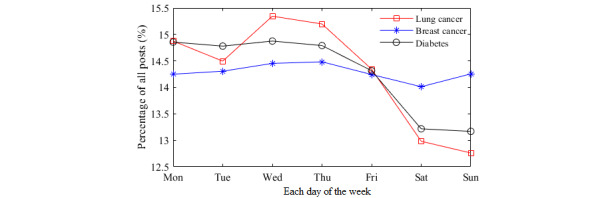
Percentage of all posts on each day of the week.

We explored key user activities on each forum. To understand user stickiness in a community, we analyzed online user activities. Community managers can adopt different strategies and incentives to improve their user experiences based on the behaviors of these users. Figure 3 shows the percentage of all posts (threads plus replies) created on each day of the week. These forums had similar trends, especially between the LCF and DCF. Besides, most online question and answer communities or vertical knowledge sharing communities are more active on weekdays than weekends, presumably due to users’ conscious work-life balance choices. By counting the frequency of posting for each day of the week by month and drawing a box plot, it can also be seen that users in the LCF and DCF post more frequently during the week and are more active during the week than on the weekend (Multimedia Appendix 1). The same conclusion was found in nononline health communities such as the DISboards [36,50] and Tianya community [15,51].

**Figure 4:**
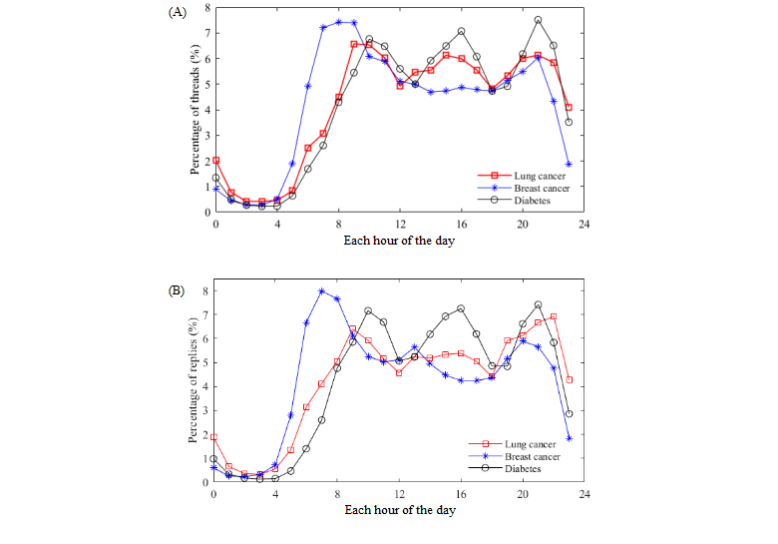
Percentage of threads (A) and replies (B) at each hour of the day.

Figure 4 shows the percentage of threads and replies created at each hour of the day. In each forum, the number of posted threads and replies increased significantly from 4 AM. In terms of the posting time of each thread, the number of posted threads in the BCF had a peak around 8 AM, which declined in the middle of the day, followed by a second peak around 9 PM. Both the LCF and DCF showed 3 peak posting moments at around 10 AM, 4 PM, and 9 PM, with their least active posting moment at around 6 PM. Furthermore, there was high similarity (*ρ*=0.927; *P*<.001) between the LCF and DCF in terms of the relative thread posting frequencies during each hour of the day. The numbers of posted threads among the 3 disease forums around 12 PM and 6 PM were less than those at other moments in the day, presumably due to the common lunch and dinner hours observed for the 2 moments of the day. Most users became active from 7 PM after dinner, and activity peaked again at 9 PM, after which the number of posted threads gradually declined as it approached bedtime. We also found that the number of posted threads in the LCF and DCF peaked around 2 or 3 hours before the Chinese mealtime (12 PM and 6 PM), likely because diabetic patients pay more attention to their diet to control blood glucose. Similarly, lung cancer affects the digestive function of patients, which may cause decreased appetite. Especially in the advanced stage of lung cancer, it is indispensable to control and adjust the diet. Therefore, most users consulted about diet in advance, leading to a significantly increased number of posted threads. Due to the different dietary behaviors of breast cancer patients and those of patients with the other 2 diseases, the active posting periods of the BCF were different from those of the other 2 forums. In terms of the posting time of replies, we found that the relative frequencies of posting for threads and replies during each hour of the day were highly positively correlated in each forum. Table 3 shows the Spearman correlation test results. Similarly, the least numbers of replies were posted at around 12 PM and 6 PM, and this was also due to their overlap with common mealtimes.

**Table 3:** Spearman rank correlation coefficients of the relative frequencies of posting for threads and replies during each hour of the day in each forum.

Forum	*ρ*	*P* value
Lung cancer forum	0.911	<.001^a^
Breast cancer forum	0.914	<.001^a^
Diabetes consultation forum	0.976	<.001^a^

^a^Significantly correlated using the significance level of .01 (2-tailed test).

### Social Network Structure

**Figure 5:**
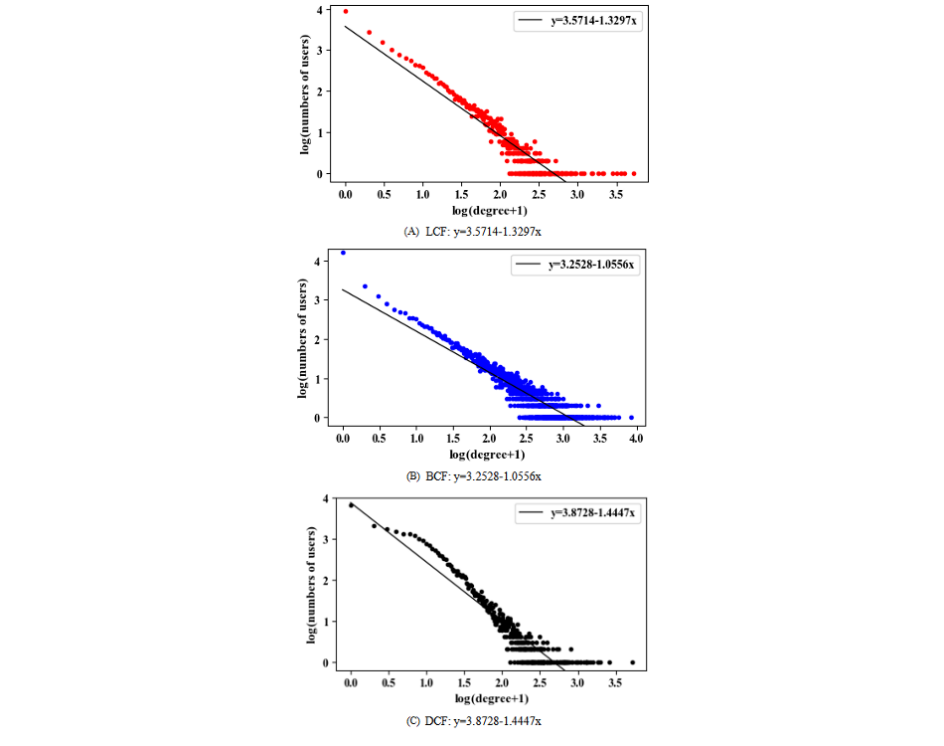
The total degree distribution of users for each of the aggregated social networks. (A) Lung cancer forum (LCF); (B) Breast cancer forum (BCF); (C) Diabetes consultation forum (DCF).

The social network structure graph visually presents the node relationship matrix of the network [41]. Table 4 shows the characteristics of the aggregated social network based on users’ reply postings on each forum. In the structure graph, each node represents a user in the community, and edges are directed links formed by replies between users. The average clustering coefficient of all 3 forums was lower than those of Facebook (0.519), Flickr (0.313), and LiveJournal (0.330) [52]. A higher global clustering coefficient indicates that there was a closer connection between users where friends tend to find each other through their mutual friends [36]. In a network, user degree is the sum of out-degree and in-degree of a user. In our data set, all users had at least one thread due to the crawling strategy used at the time of data acquisition. In the LCF, 39.7% (8968/22,610) of users had a user degree of 1; in other words, more than 39% of users only had a post. In the BCF, 51.4% (15,886/30,901) of users had a user degree of 1, and in the DCF, 24.7% (6604/26,751) of users had a user degree of 1. Most users had a relatively lower degree, while only a few users had higher degrees. Figure 5 shows the user degree distribution in each forum, from which we visually noticed that the degree distribution of each forum follows the power-law distribution. To quantitatively verify this finding, we treated the number of user degrees as an independent variable and the number of users as a dependent variable to fit a power-law curve. The resulting fitted curves were *y*=3.571*x*^−1.330^ (
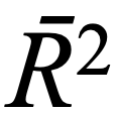
=0.9976) for the LCF, *y*=3.253*x*^−1.056^ (
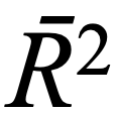
=0.9946) for the BCF, and *y*=3.873*x*^−1.445^ (
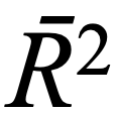
=0.9683) for the DCF. The fitting degree of the power-law curves for each forum was nearly perfect, indicating that the distribution of user degrees on each forum follows the power-law distribution and *r*>1, which further shows that the underlying social network is a typical scale-free network. Many studies have reached the same conclusions that other OHCs [20,29,53], such as Facebook [52] and Weibo [54], are also typical scale-free networks. We additionally calculated the average clustering coefficient and the average path length of the equivalent ER random networks for each forum. We randomly generated 50 sets of equivalent ER random networks, calculated σ according to equation (4), and calculated the mean value of 50 sets of σ as the final judgment coefficient. The final calculation results showed that all 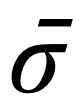 were far greater than 1 (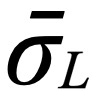
=517.15, *SD_L_*=13.31; 
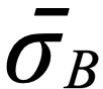
=275.23, *SD_B_*=13.02; and 
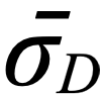
=525.18, *SD_D_*=14.38), demonstrating that all 3 forums exhibited the small-world effect.

**Table 4:** Characteristics of each aggregated social network.

Characteristic	Lung cancer forum	Breast cancer forum	Diabetes consultation forum
Number of nodes	22,610	31,909	26,751
Number of edges	183,175	739,620	223,077
Average node degree	8.10	23.179	8.34
Network diameter	10	8	11
Average clustering coefficient	0.130	0.179	0.130
Average path length	3.494	3.011	3.967
Percentage of high-degree users^a^	3.1% (697/22,610)	9.1% (2906/31,909)	2.6% (697/26,751)
Percentage of low-degree users^b^	66.6% (15,050/22,610)	67.0% (21,382/31,909)	49.3% (13,057/26,751)

^a^Percentage of users with degrees higher than or equal to 100.

^b^Percentage of users with degrees lower than or equal to 5.

Next, we analyzed the dynamic evolution characteristics of the 3 social networks from their initial establishment days (LCF: November 15, 2013; BCF: August 25, 2015; DCF: September 1, 2005) to 2020 (Multimedia Appendix 2). The results indicated that the numbers of nodes and edges increased yearly since the creation of the LCF. With the development of the community, the number of users was gradually increasing accompanied by more active user behaviors. Since the establishment of the BCF in 2015, its number of nodes has been increasing yearly, indicating that new users constantly join the forum. In the meanwhile, the number of edges in the network increased in the beginning and reached a peak in 2017, and then declined afterwards. Such a trend line shows that the disease forum reached its most active period in 2017. Since the creation of the DCF back in 2005, the numbers of nodes and edges of the forum had been steadily increasing until its peak in 2015, after which the activity of the community continuously declined, in particular between 2018 and 2019. The “sleeping rate” or “loss rate” of users in the forum was noticeable. More concretely, the number of nodes active in 2019 was 21% (943/4510) of that in 2015, while the number of edges active in 2019 was only 5% (2731/55,741) of that in 2015, and both statistics indicate an apparent recession phase of the forum.

### Analyzing UGC Using WKNs

**Figure 6:**
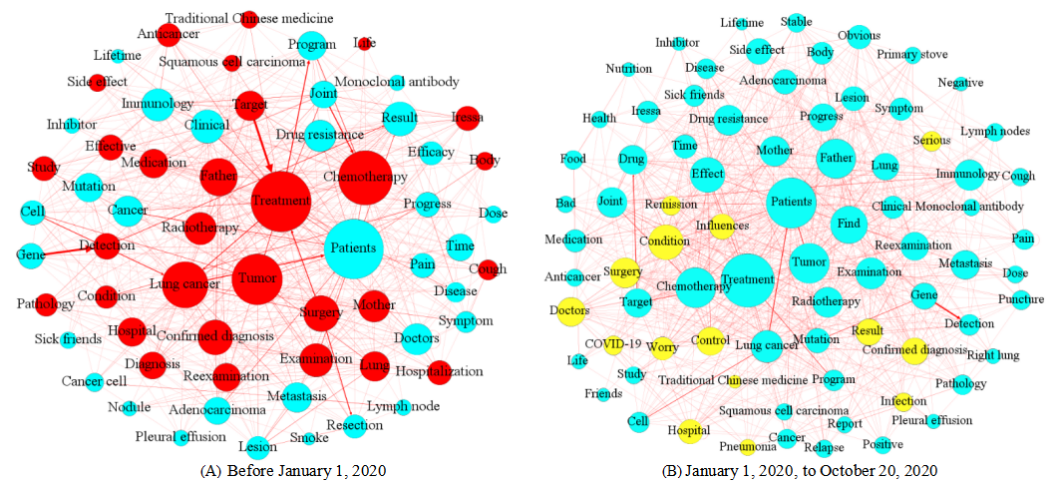
Two separate weighted knowledge networks constructed for the lung cancer forum for the analysis phases (A) November 15, 2013, to January 1, 2020, and (B) January 1, 2020, to October 20, 2020.

**Figure 7:**
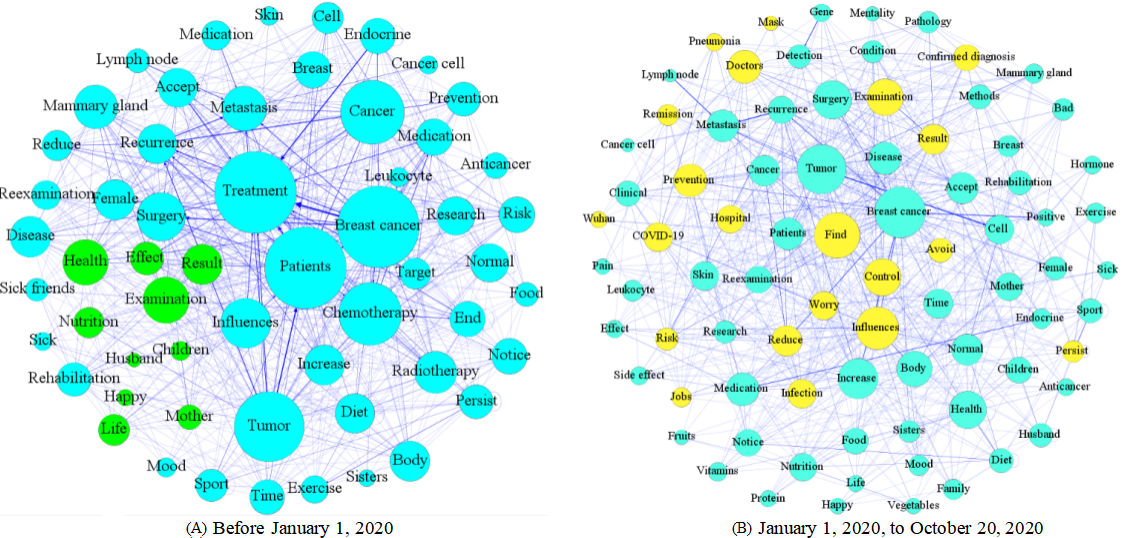
Two separate weighted knowledge networks constructed for the breast cancer forum for the analysis phases (A) August 25, 2015, to January 1, 2020, and (B) January 1, 2020, to October 20, 2020.

**Figure 8:**
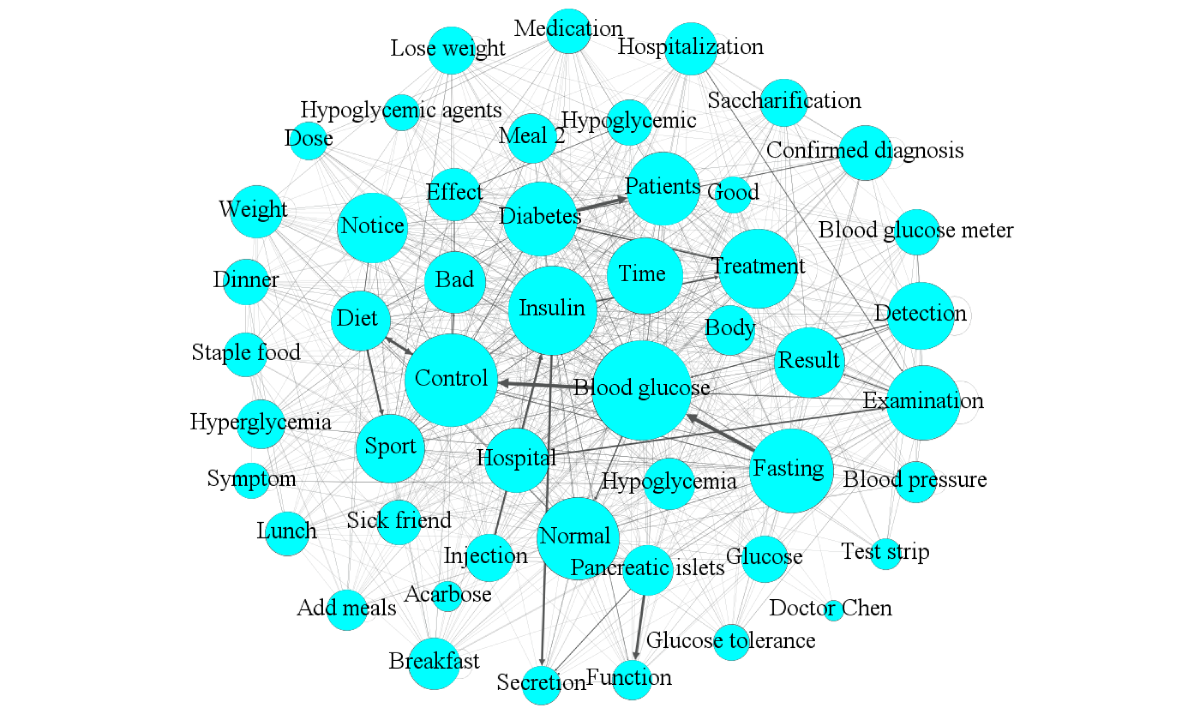
The weighted knowledge network constructed for the diabetes consultation forum during its full duration (September 1, 2005, to October 20, 2020).

Due to the COVID-19 outbreak in 2020 and its likely impact on user behaviors on OHCs, we divided our observation window into 2 periods (one before January 1, 2020, and another afterwards). By comparing user behaviors between these 2 periods, we analyzed whether the UGC changed notably due to the disease outbreak. We extracted the first 200 key phrases from the UGC of each of the 3 forums. In the preprocessing, we first filtered away keywords with no factual information according to peer literature, as well as merged synonym keywords [25,37]. For each forum, we subsequently constructed its corresponding WKN according to the construction method of the WKN model discussed above. Figures 6-8 show the resulting WKNs for each forum (Multimedia Appendix 3 for the detailed pictures). A larger node of a keyword in the WKN is associated with a greater weight of the keyword, implying more attention received by the keyword. Applying the criterion, we detected significant keywords in each forum, for example, the keywords “treatment” and “chemotherapy” in the LCF, according to Figure 6A. A darker color of the connection link between 2 keywords was associated with a higher co-occurrence frequency between these keywords. For example, the keyword “target” most frequently co-occurs with the keyword “treatment” in the LCF; thus, the connection link between these nodes has the darkest color in the forum’s corresponding WKN as shown in Figure 6A. The dense connection of a keyword indicates that the keyword co-appears with many other keywords in a sentence, for example, the keywords “treatment” and “patients” in the LCF, according to Figure 6A.

There were 8 major categories of theme feature classification strategies, namely, “etiology and pathological knowledge,” “diagnosis and examination,” “treatment,” “disease management,” “complications,” “social life,” “disease prevention,” and “education and research,” based on the classification of OHC information in PubMed literature [55]. According to these 8 classification strategies, the classification categories of the keywords of the 3 disease forums were judged, and the topic distribution was macroclassified, so as to determine the hot topics discussed in the content more clearly and quickly. There were 400 keywords in the 200 key phrases. In the LCF, the topic “disease treatment” (145/400, 36.3%) included keywords such as “treatment” and “chemotherapy;” the topic “examination and diagnosis” (118/400, 29.5%) included keywords such as “examination,” “confirmed diagnosis,” and “detection;” and the topic “social life” (38/400, 9.5%) included keywords such as “sick friends” and “life.” In the BCF, the topic “disease treatment” (128/400, 32.0%) included keywords such as “treatment” and “chemotherapy;” the topic “examination and diagnosis” (119/400, 29.8%) included keywords such as “examination” and “confirmed diagnosis;” and the topic “social life” (58/400, 14.5%) included keywords such as “sisters,” “foods,” and “sport.” In the DCF, the topic “disease treatment” (155/400, 38.8%) included keywords such as “control,” “treatment,” and “injection;” the topic “examination and diagnosis” (115/400, 28.8%) included keywords such as “examination,” “confirmed diagnosis,” and “hyperglycemia;” and the topic “social life” (31/400, 7.8%) included keywords such as “foods,” “sport,” and “sick friends.” It can be concluded that the 3 forums had “disease treatment,” “examination and diagnosis,” and “social life” themes. However, we noticed that topics related to disease prevention were rarely discussed in all 3 forums. Table 5 shows the top 10 keywords appearing in each forum, which primarily focused on disease treatment and diagnosis. Given the fact that target users of Mijian are patients after diagnosis, users of the forum tend to discuss topics on disease treatment and examination more frequently. More specifically, in both forums on Mijian (LCF and BCF) users generally paid more attention to topics on disease reexamination, metastasis, recurrence, anticancer drugs, and drug side effects. In both the BCF and DCF, users paid more attention to topics on healthy diet, exercise, and disease management.

**Table 5:** Top 10 keywords in each of the 3 forums.

Period	Lung cancer forum top keywords	Breast cancer forum top keywords	Diabetes consultation forum top keywords
Before January 1, 2020	Treatment, patients, chemotherapy, tumor, lung cancer, father, mother, confirmed diagnosis, surgery, and examination	Breast cancer, patients, treatment, tumor, chemotherapy, cancer, surgery, influences, examination, and metastasis	Blood glucose, control, insulin, fasting, treatment, normal, examination, diabetes, patients, and detection
January 1, 2020, to October 20, 2020	Treatment, patients, chemotherapy, tumor, find, father, effect, mother, lung cancer, and condition	Breast cancer, patients, treatment, tumor, chemotherapy, find, influences, increase, surgery, and examination	Blood glucose, control, insulin, fasting, treatment, normal, examination, diabetes, patients, and detection

According to Figure 6A, hot topics on the LCF before January 1, 2020, mainly concentrated on lung cancer treatment, examination and diagnosis, and social life. The most salient topic on the LCF was disease treatment, because this topic had the greatest weight. This category mainly focused on topics including keywords such as “lung cancer treatment,” “chemotherapy,” “surgical treatment,” “drug treatment,” and “treatment effect.” The most relevant topics for treatment were examination and diagnosis, including keywords such as “lung pain” and “cough symptoms,” indicating that users discussed examination and diagnostic contents, as well as the treatment of lung cancer. Ego networks consist of a focal node known as the ego, and the nodes to whom the ego is directly connected to, called alters, with edges showing links between the ego and altars or between altars. Each alter in an ego network can have its own ego network, and all ego networks combine to form the social network. The red nodes in Figure 6A formulate 2 ego networks for the keywords “father” and “mother.” We found co-occurrence relationships among the keywords “mother,” “father,” “confirmed diagnosis,” “reexamination,” and “surgery,” and they appeared in the same thread, implying that many users were probably children who consulted and communicated online health information for their parents. In the BCF, the hot topics before January 1, 2020, mainly focused on factors such as breast cancer treatment, social life, examination and diagnosis, and disease management, as shown in Figure 7A, and users mainly focused on breast cancer treatment and chemotherapy, such as endocrine therapy and treatment effects. The green nodes in Figure 7A formulate an ego network for the keyword “children.” We found the keywords related to “result,” and “health” was associated with “children.” Due to the particularity of breast cancer, patients considered some special factors such as the health status of the next generation. Different node colors were used to more clearly distinguish the ego network of a particular keyword. Since the ego networks of other nodes do not have obvious characteristic results, only the ego networks of nodes with characteristic results are discussed in the paper. In the DCF (Figure 8), the UGC mainly concentrated on topics like diabetes control and management, disease treatment, examination and diagnosis, and social life, including keywords such as “blood glucose control” and “diet control.” Meantime, the examination and diagnosis of diabetes were mostly related to disease management. In Figures 6B and 7B, yellow nodes formulate the ego network for the keyword “COVID-19,” relating to keywords such as “infection,” “remission,” “confirmed diagnosis,” and “influences.” Cancer patients represent one of the susceptible populations of COVID-19, who are more vulnerable to COVID-19 complications [56] and prone to experience severe events on exposure to COVID-19, such as admission to an intensive care unit or death. Thus, they should pay more attention to self-protection and social distancing. Since 2020, both in the LCF and BCF, users tended to mention COVID-19–related matters while discussing their medical issues. Meanwhile, the relative mention frequencies of other key topics did not change noticeably. Given that there were only a few threads (only 130 threads) posted on the DCF after 2020, no clear thematic change was observed to draw any qualitative conclusions regarding the response of its participants to COVID-19. Thus, no distinction was made.

## Discussion

### Principal Findings

This study carried out an in-depth analysis of the UGC and related online user behaviors of 3 large-scale OHCs in China. We utilized a variety of social network analysis methods and constructed a knowledge-sharing network for each OHC to study the evolution laws of the corresponding online community, discover characteristics of user behaviors, and uncover salient topics and their relations shared in the virtual community.

Since the existing research conducted on OHCs in China only examined a small-scale single disease forum, as shown in Table 1, that is, the number of data sets was less than 2000 threads and less than 10,000 replies [28,30,37,38], the scale was significantly smaller than that of analyses performed in Western countries [20,24,26], which severely undermines the reliability and comprehensiveness of the analysis findings. To meet the demand and fill the gap, we conducted thorough and extensive research on 3 representative disease forums selected from the 2 most popular Chinese OHCs. Over 80,000 users, 190,000 threads, and more than 2.8 million replies were crawled to reveal the common traits and unique characteristics of user behaviors and UGC in these forums, which can better support our findings and represent the overall characteristics of OHCs in China. The results are discussed in detail below.

First, we found that the data of these 3 disease forums were polarized, and the underlying data distributions were certainly nonuniform. In these disease forums, the number of reads per thread followed gamma distribution (*H_L_*=0, *H_B_*=0, and *H_D_*=0), and the number of replies per thread followed exponential distribution (
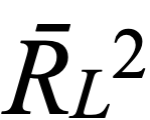
=0.946, 
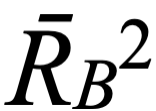
=0.958, and 
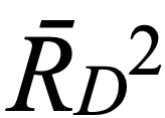
=0.971). However, the number of threads a user is involved with (
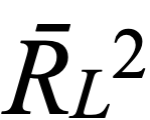
=0.978, 
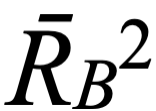
=0.964, and 
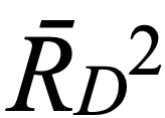
=0.970) and the number of followers of a user (
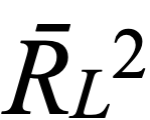
=0.989, 
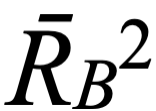
=0.962, and 
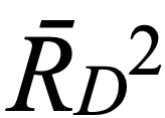
=0.990) both followed power-law distribution.

Second, users were more active during the weekdays than on weekends. The thread posting frequencies and reply frequencies in the abovementioned 3 forums had highly positive correlations between each other during each hour of the day. In particular, the LCF and DCF exhibited high temporal similarity (*ρ*=0.927; *P*<.001) in terms of the thread posting frequencies during each hour of the day. The numbers of threads and replies increased significantly from 4 AM, and the number of posted threads was relatively small in each forum around 12 PM and 6 PM. Because both lung cancer patients and diabetes patients need to pay attention to their diets, the number of posted threads in the LCF and DCF had a crest around 2 or 3 hours before the Chinese mealtime (12 PM and 6 PM).

Besides, the study showed that all 3 forums had the small-world effect (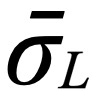
=517.15, *SD_L_*=13.31, 
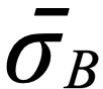
=275.23, *SD_B_*=13.02, and 
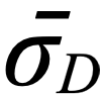
=525.18, *SD_D_*=14.38) and scale-free characteristics, and the user degrees followed the power-law distribution (
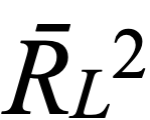
=0.997, 
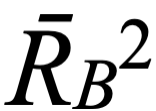
=0.994, and 
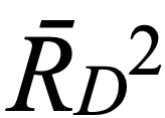
=0.968), while their global clustering coefficients were lower than those of international peer OHCs. According to the dynamic trends of the community networks, it was demonstrated that the LCF was still in the developing stage, the BCF needed to stimulate the activity of “zombie users,” and the DCF needed to attract more new users and improve the retention rate of users.

Finally, we found that several hot topics were commonly shared among the abovementioned 3 disease forums, such as disease treatment, disease examination, diagnosis, and social life. The most relevant topics for treatment were examination and diagnosis, and many children consulted related information for their parents in the LCF. In the BCF, users paid more attention to the next generation’s health, while in the DCF, users paid more attention to the detection of blood glucose and diet control. Furthermore, we noticed that in both the LCF and BCF, users tended to mention COVID-19–related matters while discussing their medical issues after the outbreak of the disease in 2020.

### Limitations

There are few limitations in this paper. On one hand, although 2 influential OHCs in China (Mijian and Sweet Home) were selected for analysis in this study, the analysis results cannot be extended to all Chinese OHCs. On the other hand, this paper only focused on the characteristics of the overall social network structure, which did not distinguish the strong and weak connections between users and user roles. At the same time, this study only analyzed the topic content of 1 user, which did not consider replies or the topic type of a thread. Therefore, subsequent research should try to add weights to the connection edges between users to study the influence of users in social networks, or study the theme changes in different periods.

### Conclusions

Our findings shed light on the basic characteristics of social networks, user behaviors, and UGC in Chinese OHCs. UGC in OHCs and related online user behaviors can be leveraged as an important source of information to gain insights on individual and population health conditions, which can be beneficial for users to understand hot topics in different forums and gain knowledge of health management. Despite the fact that OHCs are developing in China, it is indispensable to take measures to improve the retention rate and activity of users, increase user stickiness, analyze user behavior, and mine forum content themes. It is important to better mine potential content to provide users with useful information and knowledge. In conclusion, our research not only contributes to the understanding of the different characteristics of OHCs, but also helps to discover the salient topics and latent relations among these topics in each forum. Hence, effective, timely, and consistent mining and utilization of content can provide more valuable evidence for health providers and policymakers.

## Appendix

Multimedia Appendix 1 Box plot of the frequency of postings for each day of the week by month.

Multimedia Appendix 2 The dynamic evolution characteristics of social networks in different years.

Multimedia Appendix 3 The detailed pictures of Figures 6-8.

## Abbreviations

BCF: breast cancer forum
DCF: diabetes consultation forum
ER: Erdös–Rényi
LCF: lung cancer forum
NLP: natural language processing
OHC: online health community
PMI: point-wise mutual information
UGC: user-generated content
WKN: weighted knowledge network

## References

[1] Internet Plus: Premier Li’s new tech tool. The State Council of the People’s Republic of China.

[2] Maloney-Krichmar D, Preece J (2005). A multilevel analysis of sociability, usability, and community dynamics in an online health community. ACM Trans. Comput.-Hum. Interact.

[3] Open Medical and Healthcare Alliance.

[4] Action Plan for Further Improvement of Medical Services (2018-2020). National Health Commission of the People's Republic of China.

[5] Patientslikeme.

[6] MyHealthTeams.

[7] Breastcancer.org.

[8] BecomeAnEX.

[9] Manyoubang.

[10] Yi Xiang Network.

[11] Mijian360.

[12] Sweet Home Diabetes Forum.

[13] Lymphoma Home.

[14] Tianya Community.

[15] Tieba.baidu.

[16] Zhihu.

[17] Smailhodzic E, Hooijsma W, Boonstra A, Langley DJ (2016). Social media use in healthcare: A systematic review of effects on patients and on their relationship with healthcare professionals. BMC Health Serv Res.

[18] Dongxiang Z (2018). Summary of the current situation of research on the online health community in China. Intelligence Work.

[19] Wu J, Liu G, Hu X (2019). An Overview of Online Medical and Health Research: Hot Topics, Theme Evolution and Research Content. Data Analysis and Knowledge Discovery.

[20] Wang X, Zhao K, Street N (2017). Analyzing and Predicting User Participations in Online Health Communities: A Social Support Perspective. J Med Internet Res.

[21] Zhang X, Liu S, Deng Z, Chen X (2017). Knowledge sharing motivations in online health communities: A comparative study of health professionals and normal users. Computers in Human Behavior.

[22] Fernandes LDS, Calado C, Araujo CAS (2018). Social networks and health practices: influence of a diabetes online community on adherence to treatment. Cien Saude Colet.

[23] Li Y (2015). Research on Influencing Factors of Medical Information Sharing Willingness of Patients in Online Medical Community. Wanfangdata.

[24] Wang X, Zhao K, Cha S, Amato MS, Cohn AM, Pearson JL, Papandonatos GD, Graham AL (2019). Mining User-Generated Content in an Online Smoking Cessation Community to Identify Smoking Status: A Machine Learning Approach. Decis Support Syst.

[25] Jin B, Xu X (2015). Research on Theme Features in Online Health Community. Intelligence Work.

[26] Della Rosa S, Sen F (2019). Health Topics on Facebook Groups: Content Analysis of Posts in Multiple Sclerosis Communities. Interact J Med Res.

[27] Bi Q, Shen L, Evans R, Zhang Z, Wang S, Dai W, Liu C (2020). Determining the Topic Evolution and Sentiment Polarity for Albinism in a Chinese Online Health Community: Machine Learning and Social Network Analysis. JMIR Med Inform.

[28] Wu J, Zhou L (2017). The Study of Knowledge Sharing Network and Users Knowledge Interaction in Online Health Community. Information Science.

[29] Zhao K, Wang X, Cha S, Cohn AM, Papandonatos GD, Amato MS, Pearson JL, Graham AL (2016). A Multirelational Social Network Analysis of an Online Health Community for Smoking Cessation. J Med Internet Res.

[30] Shi J, Zhang B, Zhou L (2019). Research on the Dynamic Evolution of Knowledge Sharing Network of Users in Health Question and Answering Community. Information Science.

[31] Lu Y, Wu Y, Liu J, Li J, Zhang P (2017). Understanding Health Care Social Media Use From Different Stakeholder Perspectives: A Content Analysis of an Online Health Community. J Med Internet Res.

[32] Zhu Y, Guan M, Donovan E (2020). Elaborating Cancer Opinion Leaders’ Communication Behaviors Within Online Health Communities: Network and Content Analyses. Social Media + Society.

[33] Willis E, Royne MB (2017). Online Health Communities and Chronic Disease Self-Management. Health Commun.

[34] Bray F, Ferlay J, Soerjomataram I, Siegel RL, Torre LA, Jemal A (2018). Global cancer statistics 2018: GLOBOCAN estimates of incidence and mortality worldwide for 36 cancers in 185 countries. CA Cancer J Clin.

[35] Ogurtsova K, da Rocha Fernandes J, Huang Y, Linnenkamp U, Guariguata L, Cho N, Cavan D, Shaw J, Makaroff L (2017). IDF Diabetes Atlas: Global estimates for the prevalence of diabetes for 2015 and 2040. Diabetes Res Clin Pract.

[36] Li Z, Chandler H, Shen H (2018). Analysis of Knowledge Sharing Activities on a Social Network Incorporated Discussion Forum: A Case Study of DISboards. IEEE Trans. Big Data.

[37] Wu J, Wang Y, Li M, Cai S (2019). Knowledge Discovery of Online Health Communities with Weighted Knowledge Network. Data Analysis and Knowledge Discovery.

[38] Wu J, Shi L (2017). Study of the User Interaction Behavior in Online Health Community Based on Social Network Analysis. Information Science.

[39] 39 Health Network.

[40] Facebook.

[41] Social network. Wikipedia.

[42] Milgram S (1967). The Small-World Problem. Psychology Today.

[43] Watts DJ, Strogatz SH (1998). Collective dynamics of ‘small-world’ networks. Nature.

[44] Humphries MD, Gurney K (2008). Network 'small-world-ness': a quantitative method for determining canonical network equivalence. PLoS One.

[45] Barabasi AL, Albert R (1999). Emergence of scaling in random networks. Science.

[46] Loeckx D, Slagmolen P, Maes F, Vandermeulen D, Suetens P (2010). Nonrigid image registration using conditional mutual information. IEEE Trans Med Imaging.

[47] Shannon CE (1951). Prediction and entropy of printed English. The Bell System Technical Journal.

[48] Xi Y, Dang Y (2007). A Method to Represent and Measure Personal Knowledge Stocks Based on Weighted Knowledge Network. Chinese Journal of Management.

[49] Karadağ Ö, Aktaş S (2016). Goodness of fit tests for generalized gamma distribution. AIP Conference Proceedings.

[50] DISboards.

[51] Yongliang Z, Xijin T (2015). Online Behavior Analysis Based On Tianya Forum. Journal of Systems Science and Mathematical Sciences.

[52] Mislove A, Marcon M, Gummadi PK, Druschel P, Bhattacharjee B (2007). Measurement and analysis of online social networks. IMC '07: Proceedings of the 7th ACM SIGCOMM Conference on Internet Measurement.

[53] Durant KT, McCray AT, Safran C (2010). Social network analysis of an online melanoma discussion group. Summit Transl Bioinform.

[54] Luo Y, Zhou Z, Li L, Zhang H, Qin D (2015). Social Network Disciplines Analysis Based on Power-law Distribution. Computer Engineering.

[55] Zhang J, Zhao Y (2013). A user term visualization analysis based on a social question and answer log. Information Processing & Management.

[56] Guan W, Liang W, Zhao Y, Liang H, Chen Z, Li Y, Liu X, Chen R, Tang C, Wang T, Ou C, Li L, Chen P, Sang L, Wang W, Li J, Li C, Ou L, Cheng B, Xiong S, Ni Z, Xiang J, Hu Y, Liu L, Shan H, Lei C, Peng Y, Wei L, Liu Y, Hu Y, Peng P, Wang J, Liu J, Chen Z, Li G, Zheng Z, Qiu S, Luo J, Ye C, Zhu S, Cheng L, Ye F, Li S, Zheng J, Zhang N, Zhong N, He J, China Medical Treatment Expert Group for COVID-19 (2020). Comorbidity and its impact on 1590 patients with COVID-19 in China: a nationwide analysis. Eur Respir J.

