# Supplementary material for: User Behaviors and User-Generated Content in Chinese Online Health Communities: Comparative Study
Source: J Med Internet Res. 2021 Dec 15;23(12):e19183. doi: 10.2196/19183 (PMC8717137; doi:10.2196/19183)
Supplement: Multimedia Appendix 1 [file jmir_v23i12e19183_app1.docx]

Multimedia Appendix 1. Box plot of the frequency of postings for each day of the week by month.


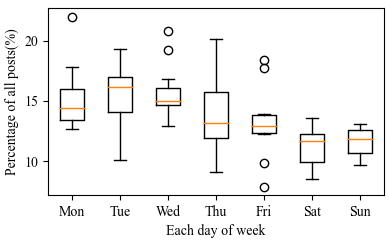


1. Lung Cancer Forum


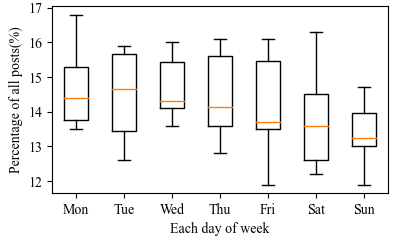


1. Breast Cancer Forum


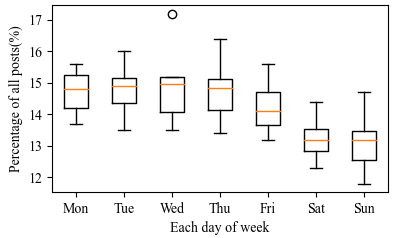


(c) Diabetes Consultation Forum
