# Supplementary material for: User Behaviors and User-Generated Content in Chinese Online Health Communities: Comparative Study
Source: J Med Internet Res. 2021 Dec 15;23(12):e19183. doi: 10.2196/19183 (PMC8717137; doi:10.2196/19183)
Supplement: Multimedia Appendix 2 [file jmir_v23i12e19183_app2.docx]

Multimedia Appendix 2. The dynamic evolution characteristics of social networks in different years.

Lung cancer forum

|  | 2014 | 2015 | 2016 | 2017 | 2018 | 2019 | 2020^a^ |
| --- | --- | --- | --- | --- | --- | --- | --- |
| Number of nodes | 1405 | 1706 | 3611 | 4638 | 6211 | 9101 | 8306 |
| Number of edges | 3182 | 9287 | 22,104 | 41,477 | 57,498 | 92,253 | 71,798 |
| Average node degree | 2.1 | 4.527 | 4.283 | 6.038 | 6.139 | 6.235 | 6.593 |
| Network diameter | 11 | 10 | 9 | 9 | 9 | 9 | 9 |
| Average clustering coefficient | 0.063 | 0.129 | 0.090 | 0.102 | 0.106 | 0.132 | 0.119 |
| Average path length | 4.667 | 3.594 | 3.499 | 3.273 | 3.340 | 3.462 | 3.449 |

2020^a^: data from 2020.01 to 2020.10

Breast cancer forum

|  | 2015 | 2016 | 2017 | 2018 | 2019 | 2020^a^ |
| --- | --- | --- | --- | --- | --- | --- |
| Number of nodes | 1247 | 6217 | 7349 | 7809 | 16,030 | 10676 |
| Number of edges | 3759 | 147,642 | 259,208 | 185,006 | 149,516 | 104,489 |
| Average node degree | 3.014 | 23.748 | 35.271 | 23.691 | 9.327 | 15.647 |
| Network diameter | 9 | 9 | 6 | 8 | 8 | 9 |
| Average clustering coefficient | 0.051 | 0.22 | 0.254 | 0.215 | 0.195 | 0.151 |
| Average path length | 3.668 | 2.845 | 2.742 | 2.903 | 3.132 | 2.849 |

2020^a^: data from 2020.01 to 2020.10

Diabetes consultation forum

|  | Number of nodes | Number of edges | Average node degree | Network  diameter | Average  clustering  coefficient | Average  path  length |
| --- | --- | --- | --- | --- | --- | --- |
| 2005 | 61 | 236 | 3.869 | 5 | 0.27 | 2.597 |
| 2006 | 435 | 1712 | 3.963 | 12 | 0.212 | 3.784 |
| 2007 | 1211 | 4961 | 4.097 | 11 | 0.184 | 3.714 |
| 2008 | 2842 | 15,258 | 5.369 | 11 | 0.152 | 3.944 |
| 2009 | 2893 | 16,547 | 5.72 | 10 | 0.149 | 3.635 |
| 2010 | 2347 | 11,252 | 4.794 | 13 | 0.113 | 4.367 |
| 2011 | 2668 | 13,554 | 5.08 | 12 | 0.098 | 4.337 |
| 2012 | 3004 | 16,755 | 5.578 | 10 | 0.091 | 3.867 |
| 2013 | 3195 | 17,531 | 5.487 | 12 | 0.097 | 3.696 |
| 2014 | 3213 | 19,244 | 5.989 | 14 | 0.086 | 4.006 |
| 2015 | 4510 | 55,741 | 12.359 | 9 | 0.149 | 3.214 |
| 2016 | 3974 | 26,625 | 6.7 | 10 | 0.098 | 3.774 |
| 2017 | 3691 | 22,561 | 6.112 | 10 | 0.074 | 4.032 |
| 2018 | 1643 | 5387 | 3.279 | 14 | 0.057 | 4.594 |
| 2019 | 943 | 2731 | 2.896 | 11 | 0.061 | 3.963 |
| 2020^a^ | 249 | 2180 | 1.463 | 4 | 0.06 | 1.682 |

2020^a^: data from 2020.01 to 2020.10
