# Supplementary material for: User Behaviors and User-Generated Content in Chinese Online Health Communities: Comparative Study
Source: J Med Internet Res. 2021 Dec 15;23(12):e19183. doi: 10.2196/19183 (PMC8717137; doi:10.2196/19183)

Multimedia Appendix 3. The detailed pictures of Figures 6-8.

Figure 6: Two separate WKNs constructed for LCF for each analysis phase (November 15, 2013-January 1,2020 and January 1,2020-October 20, 2020).


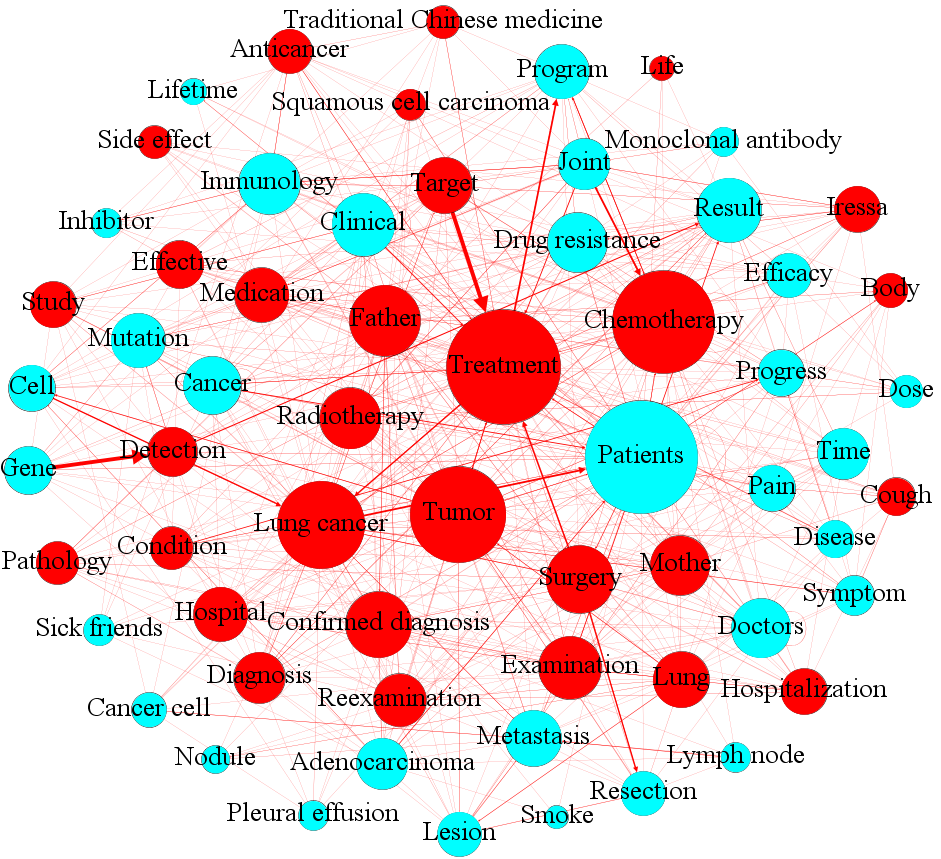
s

(a) Before January 1, 2020


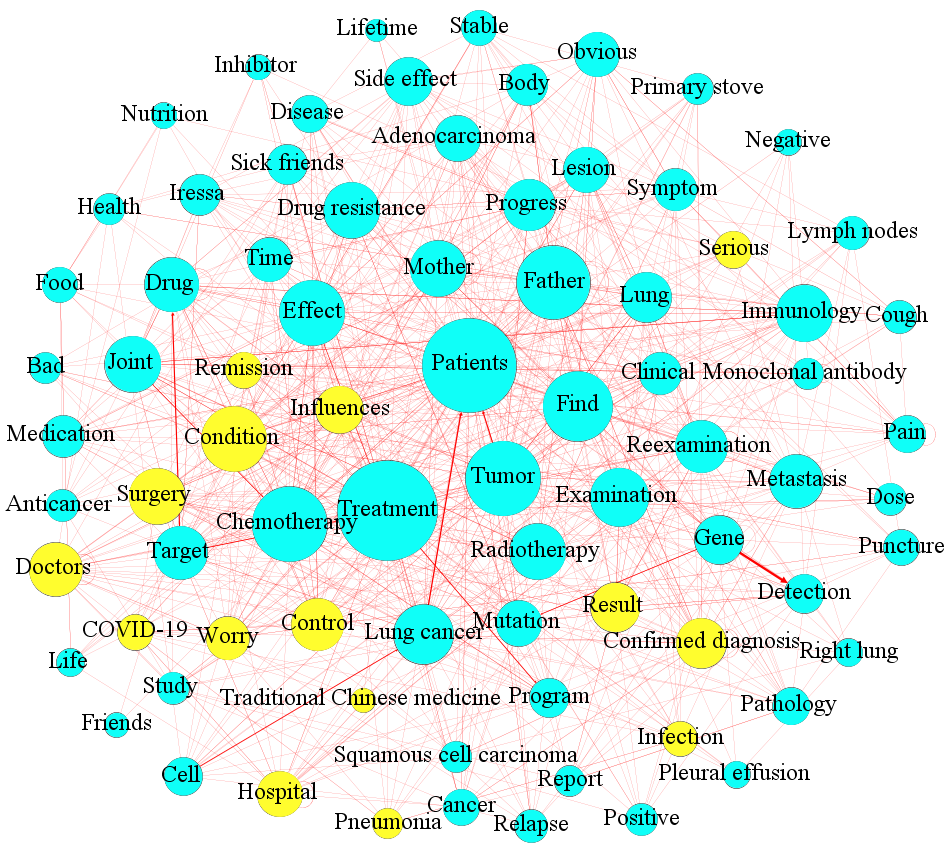


(b) January 1, 2020 to October 20, 2020

Figure 7: Two separate WKNs constructed for BCF for each analysis phase (August 25, 2015-January 1,2020 and January 1,2020-October 20, 2020).


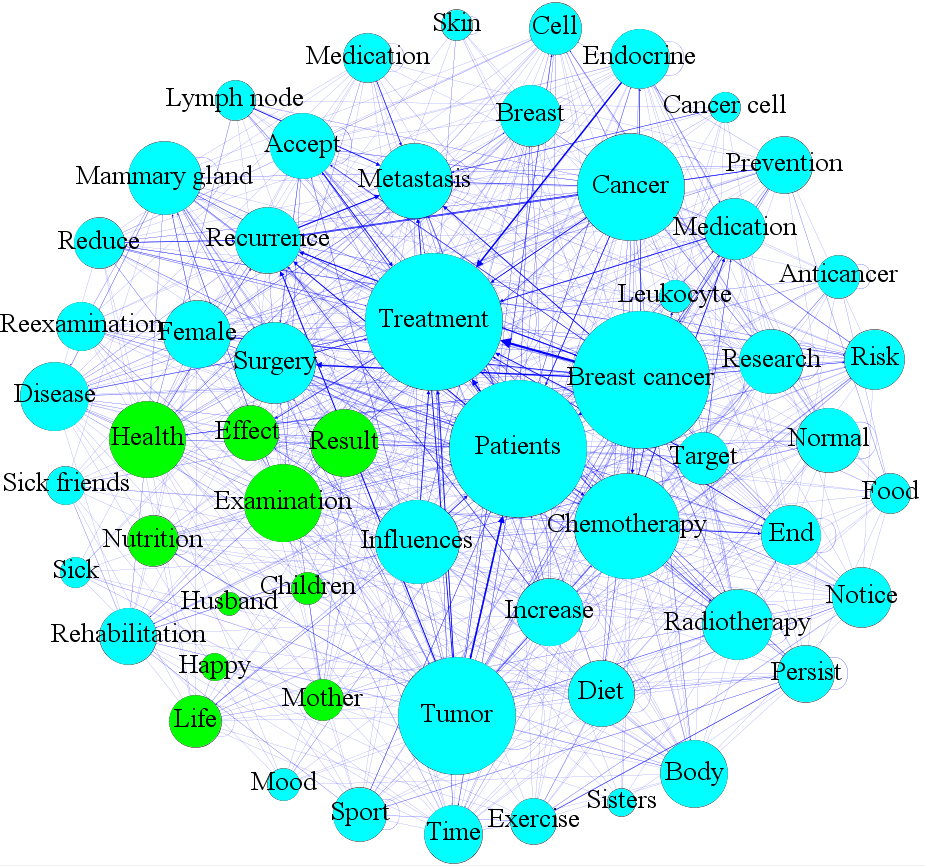


(a) Before January 1, 2020


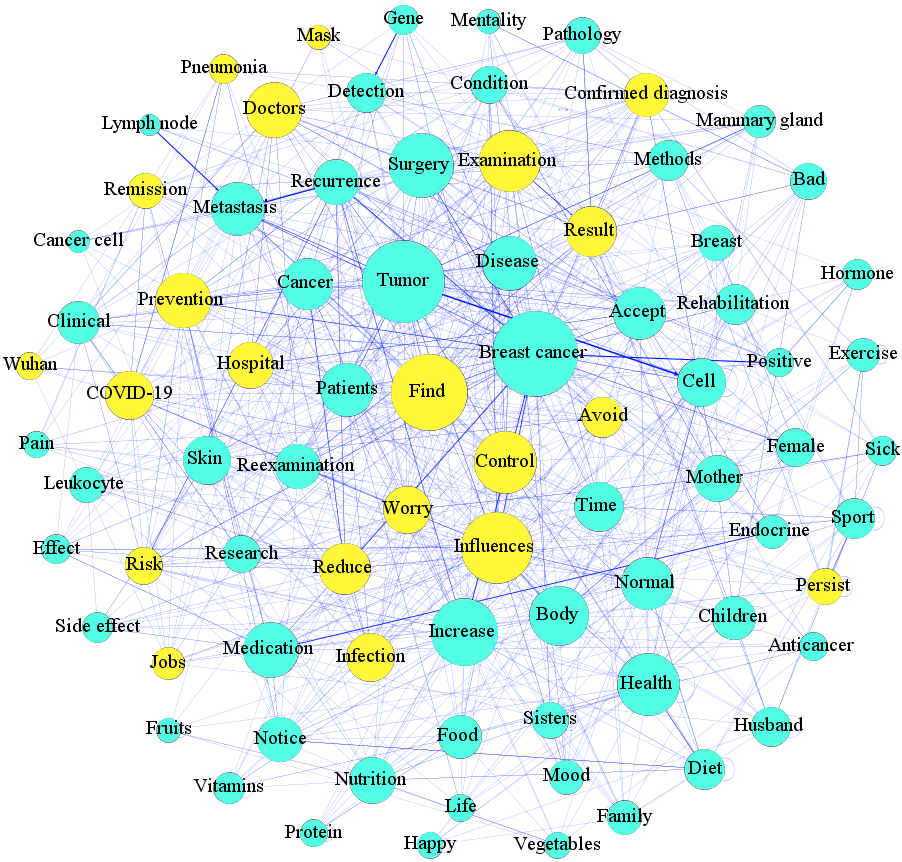


(b) January 1, 2020 to October 20, 2020

Figure 8: The WKN constructed for DCF during its full duration (September 1, 2005-October 20, 2020)


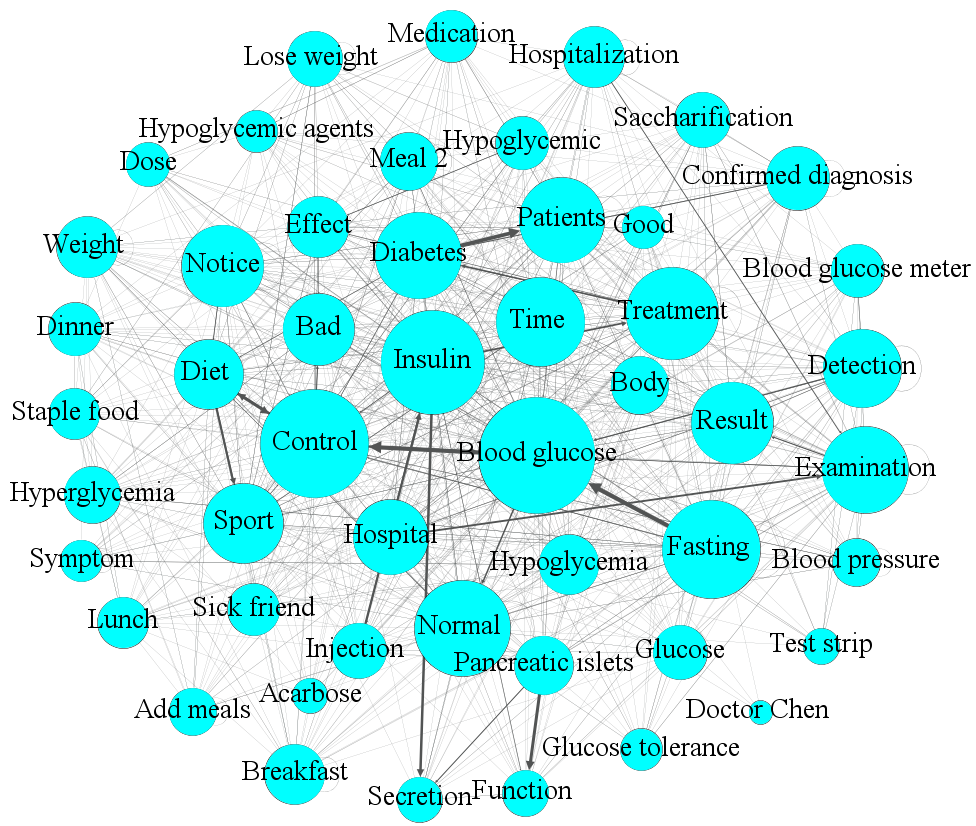

Supplement: Multimedia Appendix 3 [file jmir_v23i12e19183_app3.docx]
